# Epigenetic profiling of social communication trajectories and co-occurring mental health problems: a prospective, methylome-wide association study

**DOI:** 10.1017/S0954579420001662

**Published:** 2021-01-26

**Authors:** Jolien Rijlaarsdam, Charlotte A. M. Cecil, Caroline L. Relton, Edward D. Barker

**Affiliations:** 1 Department of Child and Adolescent Psychiatry/Psychology, Erasmus MC University Medical Center Rotterdam, Rotterdam, the Netherlands; 2 Department of Epidemiology, Erasmus MC University Medical Center Rotterdam, Rotterdam, the Netherlands; 3 Medical Research Council Integrative Epidemiology Unit, University of Bristol, Bristol, UK; 4 School of Social and Community Medicine, University of Bristol, Bristol, UK; 5 Department of Psychology, Institute of Psychiatry, Psychology and Neuroscience, King’s College London, London, UK

**Keywords:** ALSPAC, autistic traits, DNA methylation, longitudinal, methylome-wide

## Abstract

While previous studies suggest that both genetic and environmental factors play an important role in the development of autism-related traits, little is known about potential biological mechanisms underlying these associations. Using data from the Avon Longitudinal Study of Parents and Children (ALSPAC), we examined prospective associations between DNA methylation (DNAm: *n*_birth_ = 804, *n*_age 7_ = 877) and trajectories of social communication deficits at age 8–17 years. Methylomic variation at three loci across the genome (false discovery rate = 0.048) differentiated children following high (*n* = 80) versus low (*n* = 724) trajectories of social communication deficits. This differential DNAm was specific to the neonatal period and not observed at 7 years of age. Associations between DNAm and trajectory membership remained robust after controlling for co-occurring mental health problems (i.e., hyperactivity/inattention, conduct problems). The three loci identified at birth were not replicated in the Generation R Study. However, to the best of our knowledge, ALSPAC is the only study to date that is prospective enough to examine DNAm in relation to longitudinal trajectories of social communication deficits from childhood to adolescence. Although the present findings might point to potentially novel sites that differentiate between a high versus low trajectory of social communication deficits, the results should be considered tentative until further replicated.

## Introduction

Autism is associated with high health care utilization because of its persistence, long-term impairments, and high comorbidity with other psychiatric disorders (Ganz, 2007; Joshi et al., 2010; Simonoff et al., 2008). Like most complex phenotypes, autism is thought to have a multifactorial etiology that includes a dynamic interplay of genetic and environmental factors. It has been shown that autism has a strong heritable basis (64%–91%) (Tick, Bolton, Happe, Rutter, & Rijsdijk, 2016) and shares a common genetic vulnerability with other neuropsychiatric disorders such as attention-deficit/hyperactivity disorder (ADHD) (Lichtenstein, Carlstrom, Rastam, Gillberg, & Anckarsater, 2010). Environmental factors such as prenatal exposure to maternal smoking or stress also play a role in the etiology of various neuropsychiatric disorders, including autism (Ronald, Pennell, & Whitehouse, 2010; St Pourcain et al., 2011). However, little is known about the biological mechanisms through which these genetic and environmental effects on autism manifest.

Epigenetic mechanisms that regulate gene expression, such as DNA methylation (DNAm), have been shown to (a) respond to both genetic and environmental factors (Meaney, 2010) and (b) associate with various psychiatric conditions, including autism (Vogel Ciernia & LaSalle, 2016). As such, DNAm may represent a potential biological mechanism that could explain autism susceptibility across the life span. However, findings to date are mixed (Dall’Aglio et al., 2018) and may reflect the primarily clinical focus of methylome-wide studies on autism. For example, the common etiology of neuropsychiatric disorders suggests caution in relying solely on the diagnostic criteria of autism, without considering subthreshold manifestations. Core characteristics of autism, including social communication deficits, exist in all individuals to varying degrees, resulting in a dimensional distribution in the general population (Bolte, Westerwald, Holtmann, Freitag, & Poustka, 2011; Skuse, Mandy, & Scourfield, 2005).

The vast majority of methylome-wide studies have utilized clinical samples, relying primarily on clinical diagnosis of autism in case-control designs (e.g., Andrews et al., 2018; Hannon et al., 2018b). One notable exception is the recent population-based study by Massrali et al. (2019), examining the association between DNAm at birth and childhood social communication deficits – a core component of autism-related traits in childhood. Although the authors did not identify a significant association between DNAm and social communication deficits, they did observe a significant correlation with the results from a methylome-wide study of a similar childhood phenotype (i.e., pragmatic communication) measured in the same cohort. However, as this study involved measures at a single time point, longitudinal evidence of the developmental trajectories of social communication deficits is lacking. The use of developmental trajectories to describe the longitudinal course of autism-related traits may help unravel biological mechanisms for subgroups of social communication deficits that differentiate over time (Lord, Bishop, & Anderson, 2015).

The present study sought to address this issue by conducting the first epigenome-wide association study (EWAS) of developmental trajectories of social communication deficits in a large, prospective, population-based sample featuring repeated measures of both DNAm and social communication deficits spanning birth to early adolescence. These data enabled us to uniquely investigate the longitudinal course of autism-related traits in relation to genome-wide DNAm at birth and childhood. Our aim was to address the following key questions.

1. Are DNAm patterns at birth and early childhood prospectively associated with trajectories of social communication deficits (age 8–17 years)?
2. Do the identified DNAm markers associate with genetic and environmental risk exposures?
3. Are associations with DNAm unique to social communication deficits trajectories or shared with – or even entirely confounded by – other mental health symptom domains?

## Method

### Participants

Participants were drawn from the Accessible Resource for Integrated Epigenomics Studies (ARIES; Relton et al., 2015) (www.ariesepigenomics.org.uk), containing DNAm data for a subset of 1,018 mother–offspring pairs and nested within the Avon Longitudinal Study of Parents and Children (ALSPAC). ALSPAC is an ongoing epidemiological study of children born from 14,541 pregnant women residing in Avon, United Kingdom, with an expected delivery date between April 1991 and December 1992 (85% of the eligible population; Fraser et al., 2013). Ethical approval for the study was obtained from the ALSPAC Ethics and Law Committee as well as local research ethics committees. Informed consent was obtained from all ALSPAC participants. The original ALSPAC sample is representative of the general population (Boyd et al., 2013). The study website contains details of all the data available through a fully searchable data dictionary and variable search tool (http://www.bris.ac.uk/alspac/researchers/data-access/data-dictionary/). For this study, we included children from ARIES who had available data on social communication ratings (at ages 8–17 years) as well as DNAm data at birth (*n* = 804, 49% male) or at 7 years of age (*n* = 877, 50% male; 88% overlapping with the data at birth). A total of 769 children with social communication ratings had DNAm data at both time points, while a minority had DNAm data only at birth (*n* = 35) or only at age 7 (*n* = 108).

### Measures

#### Social communication deficits

Social communication skills were assessed using the 12-item Social Communication Disorder Checklist (SCDC; Skuse et al., 2005), which is a validated screening instrument of social reciprocity and verbal/nonverbal communication with high sensitivity and specificity for autism. Mother-reported SCDC scores for children and adolescents were computed at ages 8, 11, 14, and 17 years, with higher scores reflecting more social communication deficits (score range 0–24).

#### DNAm data

Array-based methylation quantification was conducted by ARIES (Relton et al., 2015). DNA was measured from cord blood drawn from the umbilical cord upon delivery and from whole blood at 7 years of age. Following extraction, 500 ng of genomic DNA was bisulfite-converted using the EZ-DNA methylation kit (Zymo Research, Orange, CA). DNAm was quantified using the Illumina HumanMethylation450 BeadChip (Illumina, San Diego, CA) according to the standard protocol. The arrays were scanned using an Illumina iScan (software version 3.3.28). Initial data quality control was conducted using GenomeStudio (version 2011.1, Illumina). For further details on the DNAm preprocessing pipeline see the Supplementary Material, section 1.

Samples (*n*_birth_ = 25; *n*_age 7_ = 8) or probes (*n*_birth_ = 7873; *n*_age 7_ = 4861) that failed quality control employed by the ARIES team (>1% probes/samples with background detection *p* value ≥ .05) were excluded from further analysis. Furthermore, multiple checks for sample mismatch were carried out. Specifically, samples were checked by calculating concordance with (a) genome-wide association data from the same participants, (b) single nucleotide polymorphism (SNP) probes on the Illumina 450K array across mother and child, and (c) sex. Data were quantile normalized using the *dasen* function as part of the wateRmelon package (wateRmelon_1.0.3; Pidsley et al., 2013) within the R statistical analysis environment.

We removed probes previously reported to be cross-reactive or polymorphic (Chen et al., 2013; Price et al., 2013), in addition to SNP (i.e., “rs”) probes (total probes removed = 72,068). We further removed participants with non-Caucasian or missing ethnicity (based on self-reports; *n* = 61). This left a total of 397,879 probes and 828 samples (cord blood at birth) and 397,791 probes and 903 samples (blood at age 7 years) after quality control.

For each probe, DNAm levels were indexed by beta values (i.e., the ratio of methylated signal divided by the sum of the methylated and unmethylated signal [*M*/(*M* + *U* + 100)], ranging from 0 (no methylation) to 1 (complete methylation). Probes were annotated using the Illumina HumanMethylation450 BeadChip annotation file.

#### Risk exposures

As risk exposures, we included prenatal maternal smoking and exposure to stress. Maternal smoking was measured during the first trimester of pregnancy via maternal ratings, using a dichotomous (12.2% yes) variable. With regards to prenatal stress exposure, we included a cumulative risk score of prenatal (18–32 weeks) adversity. This score was based on 56 dichotomous items from the Life Events Inventory (adapted in ALSPAC based on the work of Barnett, Hanna, & Parker (1983) and Brown, Sklair, Harris, & Birley (1973)) and the Family Adversity Index (Bowen, Heron, Waylen, & Wolke, 2005; Wolke, Steer, & Bowen, 2004), which are widely used (e.g., Barker, Oliver, Viding, Salekin, & Maughan, 2011; Mesirow, Cecil, Maughan, & Barker, 2017). Briefly, the items were organized into four conceptually distinct but related risk domains, and summed to create the following cumulative scores covering the following four domains: (a) life events (e.g., death in family, accident), (b) contextual risks (e.g., poor housing, financial problems), (c) parental risks (e.g., psychopathology, criminal behavior), and (d) interpersonal risks (e.g., partner abuse, family conflict). The overall cumulative risk score was estimated using confirmatory factor analysis, as described elsewhere (Cecil et al., 2014; Rijlaarsdam et al., 2016). For the full list of items contained in these cumulative scores, see Cecil et al. (2014).

#### Child characteristics

We assessed autism-related phenotypes using Pupil Level Annual School Census (PLASC) records and the Development and Well-Being Assessment (DAWBA; Goodman, Ford, Richards, Gatward, & Meltzer, 2000). The UK Department for Children, Schools and Families has a database referred to as PLASC, to which ALSPAC data are routinely linked. It encompasses all pupils who pass through the state-maintained educational system in the UK, providing data on children with special educational needs (www.dcsf.gov.uk). The DAWBA is a validated semi-structured interview. Parents complete open and closed questions about a range of symptoms relevant to youth psychiatric disorders. This study used the DAWBA *Diagnostic and Statistical Manual of Mental Disorders*, fourth edition (DSM-IV) diagnosis of pervasive developmental disorder at 7 years of age.

Child mental health symptoms were assessed using the Strengths and Difficulties Questionnaire (SDQ; Goodman, 2001), which is a widely used screening questionnaire with high reliability and validity. We included mothers’ reports on their children’s conduct problems (e.g., “often fights with other children or bullies them”), hyperactivity/inattention (e.g., “restless, overactive, cannot stay still for long”), emotional difficulties (e.g., “often unhappy, downhearted or tearful”), and peer problems (e.g., “rather solitary, tends to play alone”). For each subscale, scores were combined across three time points – at age 8 (*n* = 734), 10 (*n* = 755), and 13 (*n* = 719) – using confirmatory factor analysis (see Table S1 in the Supplementary Material for descriptive statistics and factor loadings). Child IQ was assessed at 8 years of age using the Wechsler Intelligence Scale for Children (WISC-III; Wechsler, 1991).

### Statistical analysis

#### Social communication deficits trajectories

Developmental trajectories of social communication deficits were modeled through longitudinal latent profile analysis as implemented within Mplus (version 7.11; Muthén & Muthén, 2011). This type of analysis aims to identify a latent class variable that corresponds to different developmental patterns of social communication deficits across multiple time points (e.g., high vs. low levels at ages 8–17 years). A series of models was fitted, beginning with a one-class model and moving to a four-class model. In line with previous research documenting both latent profile and methylome-wide analyses (Barker et al., 2018), model selection was captured through (a) entropy, with values closer to 1 suggesting a higher model classification accuracy, and (b) the overall percentage of children estimated in the different trajectories. Currently, to our knowledge, there is no software program that can both estimate mixture models and perform bias-adjusted methods within a methylome-wide association analysis. Hence, as we estimated the trajectories in Mplus and then ran the methylome-wide association analyses in R, we paid close attention to potential bias that could affect confidence in the results. According to a simulation study using ALSPAC data (Heron, Croudace, Barker, & Tilling, 2015), there is little detriment to assessing covariates (i.e., low bias) when using a classification approach with high levels of entropy and high class-separation (i.e., low overlap in class membership).

We further assessed group contrasts for child characteristics including autism-related phenotypes, mental health symptoms, and IQ. These group contrasts indicated the descriptive statistics of given variables with the likelihood of trajectory membership relative to the contrast group.

#### Methylome-wide analysis of social communication trajectories

Methylome-wide analysis (DNAm beta values at birth and at 7 years of age) of social communication trajectories was performed in R version 3.4.3 (R Core Team, 2017) using the CpGassoc package (Barfield, Kilaru, Smith, & Conneely, 2012). Analyses were conducted using linear regression adjusting for sex, sample plate, and estimates of cell type proportions (CD8T lymphocytes, CD4T lymphocytes, natural killer cells, B lymphocytes, and monocytes), as detailed by Houseman et al. (2012). Differentially methylated probes (DMPs) were considered statistically significant if they passed a false discovery rate (FDR) correction of *q* < 0.05. For these DMPs, we additionally examined the extent to which DNAm levels at birth correlated with those at age 7 (i.e., autocorrelations or temporal stability). The probes were winsorized prior to analyses to reduce the influence of potential outliers (>3 *SD*).

#### Genetic and environmental influences underlying the top hits

We then investigated to what extent identified associations were explained by genetic factors or known prenatal environmental risks, including maternal smoking and stress exposure. Smoking is particularly important to account for, being one of the most consistently associated exposures to DNAm alterations (e.g., see Hannon et al., 2019).

As our sample was underpowered to directly examine genetic effects, we used the ALSPAC-derived methylation quantitative trait loci database (mQTLdb) resource (http://www.mqtldb.org/; Gaunt et al., 2016) to search for known mQTLs associated with our DMPs. This mQTLdb characterizes genome-wide significant *cis* effects (i.e., SNP within ±1,000 base pairs of the DNAm site) and *trans* effects (i.e., ±1 million base pairs) on DNAm levels across Illumina 450K probes at five different life stages, including cord blood DNAm at birth (Gaunt et al., 2016). We used mQTLdb results from the conditional genome-wide complex trait analysis (GCTA) set to identify mQTLs with the most representative, independent effect on each DNAm site in order to account for linkage disequilibrium (Gaunt et al., 2016). We then examined the associations between prenatal environmental risk exposures and DMPs, using linear regression correcting for sex, sample plate, and cell type.

#### Univariate and multivariate models: mental health symptoms

To assesses whether potentially co-occurring mental health problems (conduct problems, hyperactivity/inattention, emotional difficulties, peer problems) confound links between social communication deficits and DNAm, we examined their associations with the DMPs. To this end, we used both univariate and multivariate linear regression, correcting for sex, sample plate, and cell type. The multivariate linear regression model was designed to examine whether epigenetic associations are unique to social communication deficits trajectories or shared with – or even entirely confounded by – other mental health symptom domains.

#### Replication

We used data from the Generation R Study (Kooijman et al., 2016) to examine the replicability of our initial observations in ALSPAC. The Generation R Study is a population-based prospective cohort study from fetal life onwards. Pregnant women residing in Rotterdam, the Netherlands, with expected delivery dates between April 2002 and January 2006 were invited to participate. The study was approved by the Medical Ethical Committee of the Erasmus MC University Medical Center Rotterdam, and written consent was obtained for all the participants. The Generation R Study Biobank has DNAm results from 1,396 cord blood samples measured using the Illumina 450 Infinium BeadChip (Kruithof et al., 2014).

In the Generation R Study, children’s social responsiveness was assessed via parental ratings using an 18-item short form of the Social Responsiveness Scale (SRS; Constantino et al., 2003) when children were 6 years of age. In this study, the subscale indexing social communication deficits (eight items, e.g., “slow or awkward in interactions with peers”) was used. Items were rated on a 4-point scale, ranging from 0 (= *never true*) to 3 (= *almost always true*). In the absence of the repeated measures that are required for latent profile analysis, we used the social communication score at age 6 years both continuously and categorically (mean + *SD*). To our knowledge, ALSPAC is the only cohort that is prospective enough to examine the association between neonatal DNAm patterns and trajectories of social communication difficulties from childhood to adolescence.

DMPs were extracted from the Generation R epigenome-wide data set and regressed on the social communication score, using linear regression correcting for sex, sample plate, and cell type (Houseman et al., 2012).

## Results

### Social communication deficits trajectories

**Figure 1.**
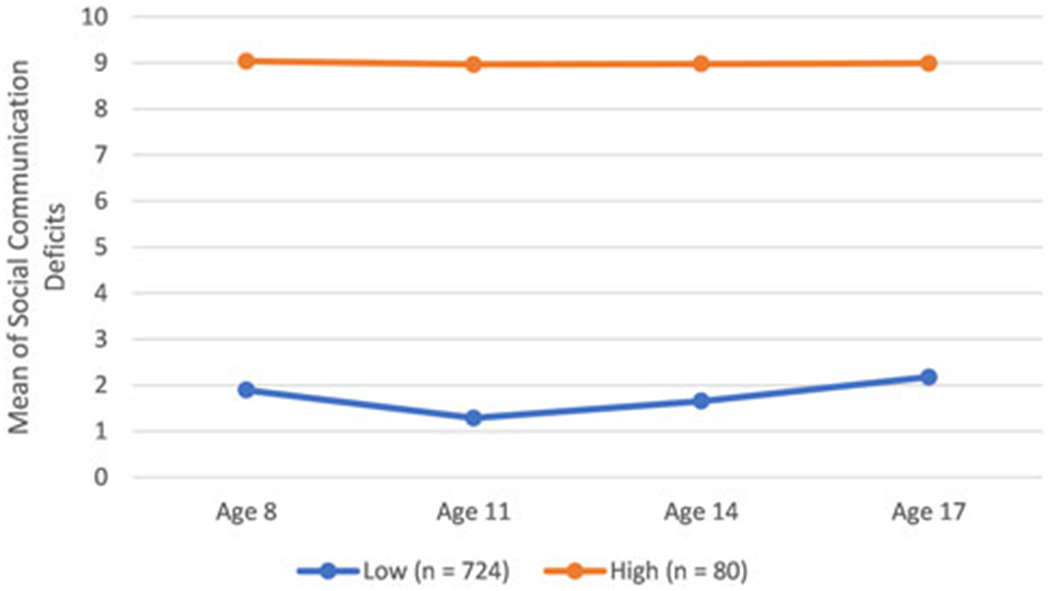
Trajectory of social communication deficits.

Latent profile analyses yielded a two-trajectory solution (Figure 1) for social communication problems, showing clearly discernible “high” (10%, *n* = 80) and “low” (90%, *n* = 724) groups between ages 8 and 17 years (entropy = 0.955). Table S2 of the Supplementary Material shows that the separation of the two-trajectory model was better than that of the three-trajectory (entropy = 0.916) and above models, where further trajectories were often additional low trajectories, which can result in high overlap of trajectory contrasts. Furthermore, the three-trajectory model (and above) resulted in a much smaller percentage of children in the high trajectory (3.5%), challenging methylome-wide analyses.

Social communication deficits group contrasts for this two-trajectory solution are displayed in Table 1. There were no sex differences in the likelihood of high (58.8% boys) versus low (47.7% boys) trajectory membership, *p* = .059. Regarding the autism-related phenotypes, records from the UK Department for Children, Schools and Families (PLASC, *n* = 668) did not indicate the presence of autism disorder in our study sample. Only one child (total *n* = 764) met criteria for a DAWBA-based DSM-IV diagnosis of pervasive developmental disorder, again hampering group comparisons. Group contrasts for conduct problems, hyperactivity/inattention, emotional difficulties, and peer problems were all significant and not specific to any individual domain (all *p* ≤ 0.001, see Table 1). IQ did not differ between the groups (*p* = .215).

### Methylome-wide analysis of the social communication trajectories

At birth, three probes were associated with low versus high social communication deficits trajectories after genome-wide correction (*q* < 0.05; Table 2). The full EWAS results of DNAm collected at birth are available in the Zenodo repository (https://doi.org/10.5281/zenodo.4031357) and the EWAS Catalog (http://www.ewascatalog.org/). The mean percent methylation difference between the high-and low-trajectory groups for the three DMPs identified at birth ranged from 3% to 4% (Table 2), with small effect sizes consistent with what has been reported in the wider psychiatric epigenetic literature (Barker et al., 2018; Walton et al., 2017). Of the DMPs, both cg23234820 and cg04112471 were hypomethylated in the high social communication deficits trajectory, whereas cg06825512 was hypermethylated. Cg23234820 is annotated to *A4GALT* (Alpha 1,4-Galactosyltransferase (P Blood Group)), a gene involved in the biosynthesis of the P(k) blood antigen (Iwamura et al., 2003). Cg06825512 is annotated to *APCDD1* (APC Down-Regulated 1), an inhibitor of the Wnt signaling pathway (Shimomura et al., 2010) that has previously been linked to autism (Cusco et al., 2009). Cg04112471 is annotated to *SPSB4* (SplA/Ryanodine Receptor Domain And SOCS Box Containing 4), a gene regulating EphB2-dependent cell repulsive responses (Okumura et al., 2017) that has also been associated with autism (Wang et al., 2014).

With regards to temporal stability, we first tested, for each DMP, the extent to which DNAm levels at birth correlated with those at age 7 (i.e., autocorrelations). Only cg06825512 showed a significant (*p* < .05) autocorrelation, which was in the positive direction (*r* = .27). However, none of the DMPs identified at birth continued to prospectively associate with social communication deficits trajectories by age 7 (at FDR corrected [*q* < 0.05] and even nominal threshold [*p* < .05]). More generally at age 7, we identified no genome-wide corrected DMPs between low versus high social communication deficits trajectories. The full EWAS results of DNAm collected at age 7 are available in the Zenodo repository (https://doi.org/10.5281/zenodo.4031387) and the EWAS Catalog (http://www.ewascatalog.org/).

### Genetic and environmental influences underlying the top hits

The three DMPs identified at birth were carried forward to explore associations with genetic and environmental risk. Based on a mQTLdb search, we found that none of the DMPs were associated with genetic variations known to have an effect on DNAm levels. This lack of mQTLs seems to be supported by a heritability tool characterizing additive genetic influences as opposed to shared and nonshared environmental influences on DNAm, based on data from monozygotic and dizygotic twins (Hannon et al., 2018a). Both cg23234820 and cg04112471 showed low additive genetic influences (*r* = 4.08E^−12^ and *r* = 9.58E^−15^, respectively), whereas cg06825512 showed moderate genetic influences (*r* = 0.64). However, as shown in Table 3, the associations between the two environmental risk exposures (i.e., prenatal stress exposure and smoking) and the three DMPs were generally weak and nonsignificant after Bonferroni correction for multiple testing (corrected *p* value = .05/three DMPs × two risk factors = 0.0083).

### Univariate and multivariate models: mental health symptoms

As can be seen in Table 3, the univariate models showed that peer problems and emotional difficulties were unrelated to all three DMPs after Bonferroni correction for multiple testing (corrected *p* value = .05/three DMPs × four mental health scores = 0.0042). However, conduct problem scores associated with cg06825512 (β = 0.12, *p* < .001), while hyperactivity/inattention scores associated with cg04112471 (β = −0.12, *p* < .001). The multivariate models showed that social communication deficits trajectories remained significantly (corrected *p* value = .05/three DMPs = 0.0167) associated with cg06825512 (β = 0.14, *p* < .001) after potentially co-occurring conduct problems were accounted for. Similarly, controlling for hyperactivity/inattention left the association between social communication deficits trajectories and cg04112471 (β = −0.15, *p* < .001) essentially unchanged.

### Replication

The results from the EWAS in ALSPAC did not replicate in the Generation R sample (see Table 4). Of the three DMPs that were associated with social communication deficits trajectories (8–17 years) in ALSPAC, two were associated with the continuous score of social deficits (6 years) in Generation R with the same direction of effect. One of the three DMPs identified in ALSPAC was associated with the categorical (mean + *SD*) score of social communication deficits in Generation R with the same direction of effect. None of them reached statistical significance.

## Discussion

Using longitudinal data spanning gestation to early adolescence, this study examined the extent to which genome-wide DNAm patterns (at birth and at 7 years of age) prospectively associate with trajectories of social communication deficits (at 8–17 years). We highlight here three key findings: (a) three loci at birth were differently methylated between high and low trajectories of social communication deficits in a temporally specific way; (b) none of the loci identified at birth associated with known mQTLs or prenatal maternal risk exposure; (c) the observed links between DNAm and social communication deficits trajectories were not accounted for by co-occurring mental health problems

Our genome-wide analysis showed that epigenetic variation across three loci at birth differed between the high versus low trajectories of social communication deficits. These loci were annotated to *A4GALT, APCDD1*, and *SPSB4* – genes involved in the biosynthesis of the P(k) blood antigen, the Wnt signaling pathway, and EphB2-dependent cell repulsive responses, respectively. Interestingly, both *APCDD1* and *SPSB4* have previously been linked to autism spectrum disorder in clinical settings (Cusco et al., 2009; Wang et al., 2014). For example, *SPSB4* was found to be similarly hypomethylated in a cross-sectional study of 131 children (age 3–12 years) diagnosed with autism spectrum disorders versus controls (Wang et al., 2014). Besides this study, the two that are most comparable to ours in terms of sample size, time point (i.e., DNAm at birth), analytical methods (i.e., methylome-wide analysis), and design (i.e., prospective study) found no significant associations between DNAm and autism (Hannon et al., 2018b; Massrali et al., 2019). Specifically, Hannon et al. (2018b) found no significant DNAm differences between patients diagnosed with autism spectrum disorder (*n* = 629) and healthy controls (*n* = 634). Similarly, Massrali et al. (2019) did not identify significant CpGs associated with social communication deficits (age 8 years). However, using DNAm data for autism from postmortem brain tissues (vs. peripheral data), the authors identified a significant concordance in effect direction of top hits in the social communication deficits EWAS. The current study is the first to investigate DNAm underlying longitudinal trajectories of social communication deficits (age 8–17 years). These trajectories are useful in that they provide us with a longitudinal case-control comparison that is based on dimensional, repeated measures of core characteristics of autism. The current results emphasize the advantage of using trajectories as autism-related phenotypes, especially in population-based samples where the prevalence of autism might be too small for meaningful methylome-wide analyses. By identifying two trajectories (high vs. low) with high entropy and high class-separation (i.e., low overlap in class membership), our study should provide results reasonably close to the best possible scenario in terms of latent profile analysis (Heron et al., 2015).

The EWAS associating DNAm at age 7 with social communication deficits trajectories revealed no genome-wide significant associations. The finding that none of the three DMPs identified at birth continued to associate with social communication deficits trajectories by age 7 is consistent with previous longitudinal epigenetic studies investigating other psychiatric phenotypes (ADHD, conduct problems, substance-use risk) in the ALSPAC cohort (Cecil et al., 2016; Rijlaarsdam et al., 2017; Walton et al., 2017) and the larger Pregnancy and Childhood Epigenetics (PACE) consortium (Neumann et al., in press). The low and mostly nonsignificant autocorrelations (birth, age 7) among the three CpGs support another ALSPAC-based study reporting low genome-wide continuity in DNAm patterns across development (Gaunt et al., 2016). Given that sample sizes were comparable across the two time points, with 88% participants represented at both time points, the temporally specific associations observed in our study might be explained by several reasons other than differences in statistical power. A possible explanation that cannot be ruled out is that temporal differences reflect tissue-specific DNAm patterns, as data were extracted from cord blood at birth and whole blood at age 7 (Bakulski et al., 2016). An alternative explanation is that the neonatal period represents a particular window of vulnerability for DNAm patterns and social communication deficits. That is, in light of the dynamic nature of both environmental and epigenetic factors, differences in DNAm patterns at birth and age 7 may reflect (a) the specific timing of environmental influences (Richmond et al., 2015) or (b) DNAm patterns at birth that induce long-lasting developmental changes but are not necessarily maintained over time (Numata et al., 2012). It is possible that, while epigenetic effects early in life may be stronger during early development (as observed in our study), weaker effects may still be observed later in childhood, particularly within high-risk clinical samples. However, given that this is the first longitudinal epigenome-wide study of trajectories of autism-related traits, we still know little about what triggers continuity versus discontinuity in epigenetic patterns (Dall’Aglio et al., 2018). Hence, the above explanations remain inevitably speculative and necessitate further investigation.

None of the DMPs identified at birth associated with genetic variations known to have an effect on DNAm levels (mQTLs; Gaunt et al., 2016). This lack of mQTLs seems to be supported by twin heritability data (Hannon et al., 2018a) showing that methylomic variation of the DMPs was primarily explained by environmental as opposed to genetic factors. However, the three DMPs were unrelated to prenatal cumulative stress exposure and smoking after multiple testing correction. Environmental effects on these DMPs may be explained by exposures other than those assessed in the current study. Furthermore, associations with environmental factors may be dependent on genetic factors. Recent evidence suggests that interactions between genotype and environmental exposures might explain more variation in DNAm than environmental exposures alone (Czamara et al., 2019; Teh et al., 2014). These Gene × Environment interactions, potentially accounting for variation in DNAm underlying social communication deficits trajectories, warrant investigation in larger studies with more statistical power.

Children following the high versus low trajectory of social communication deficits presented with more conduct problems, hyperactivity/inattention, emotional difficulties, or peer problems. Both conduct problems and hyperactivity/inattention associated with one of the three DMPs identified at birth. These results are of interest, given that early social–cognitive abilities have been implicated in the emergence and maintenance of conduct problems (Gilmour, Hill, Place, & Skuse, 2004; Oliver, Barker, Mandy, Skuse, & Maughan, 2011), with deficits for early-onset persistent conduct problems especially marked (Oliver et al., 2011). Despite the strong overlaps of social communication deficits trajectories with conduct problems and hyperactivity/inattention, controls for these mental health problems had little impact on the associations between the trajectories and DMPs. This result suggests that the observed links between DNAm and social communication deficits trajectories are not simply a consequence of co-occurrence with conduct problems or hyperactivity/inattention.

Although the three DMPs identified in ALSPAC were unrelated to social communication deficits in the Generation R Study, this lack of replication might partially reflect differences in how the phenotype was operationalized between cohorts. ALSPAC was unique in that repeated assessments of social communication deficits were collected from age 8 to 17 years, enabling the estimation of longitudinal developmental trajectories (i.e., a person-centered approach). In contrast, in the Generation R Study, social communication deficits were measured only at one time point (age 6 years) and thus examined continuously and dichotomously (i.e., a variable-centered approach). According to a recent genome-wide analysis on major depressive disorder, a more strictly defined phenotype (i.e., standardized lifetime diagnosis) captures the underlying biological mechanisms better than minimal phenotyping (i.e., phenotype definitions based on self-reports) (Cai et al., 2020). As such, trajectory-based approaches may provide us with a longitudinal case-control comparison that better captures epigenetic signal underlying general psychopathology. However, this will need to be investigated more systematically as more cohorts become available with longitudinal data spanning childhood to adolescence.

It is important to bear in mind a number of limitations while interpreting the results of this study. First, the Illumina HumanMethylation450 BeadChip covers less than 2% of all CpG sites in the genome. Thus, differentially methylated loci significantly associated with social communication deficits trajectories may be located in areas outside of the array. Second, because DNAm is tissue-specific, observations in the peripheral blood may not reflect other tissues of interest (e.g., the brain, assuming that we are looking at epigenetic changes associated to autism-related traits) that are unavailable for population-based studies of living individuals. Using the Blood–Brain Epigenetic Concordance tool (Edgar, Jones, Meaney, Turecki, & Kobor, 2017) and the Iowa Methylation Array Graphing for Experimental Comparison of Peripheral Tissue & Gray Matter (IMAGE-CpG) tool (Braun et al., 2019), we found no evidence that DNAm levels of our three DMPs correlate between blood and brain samples. However, these correlations tag specific brain areas in individuals (mostly adults) with unclear social communication deficits histories. Because DNAm patterns vary across brain regions and we do not have access to the most relevant regions to our study (e.g., amygdala), it will be important in future to establish the extent to which our findings reflect associations in the brain. The integration of transcriptomic data will also be important for assessing the functional relevance of DNAm changes to gene expression. Third, the study focused exclusively on DNAm. Other epigenetic processes (e.g., histone modifications) are likely to be important in the etiology of autism-related traits. Fourth, the exclusion of individuals with non-European ethnicity based on self-report (*n* = 61) does not imply that additional sources of unwanted variation linked to genetic ancestry are accounted for. Fifth, identifying subgroups of children with distinct trajectories using latent profile analysis is only one way of looking at longitudinal data of social communication deficits. For example, if interested in the longitudinal change in social communication deficits, one could use a mixed model approach to examine changes of the direction of the effect associated with age (see, e.g., Simpkin et al., 2015). Finally, the analyses were correlational in nature and, hence, causality cannot be inferred. The present results should be considered hypothesis-generating and in need of replication.

In summary, the present findings lend potentially novel insights into epigenetic patterns that might differentiate between high versus low trajectories of social communication deficits from childhood to adolescence, pinpointing temporally-specific markers for further interrogation. These associations were not replicated in an independent cohort. However, given that our sample was unique in that person-centered developmental trajectories could be modeled, this lack of replication might partially reflect biological heterogeneity underlying different phenotype operationalizations. The current findings should be viewed as preliminary until they have been further replicated.

## Supplementary Material

1

## Figures and Tables

**Table 1.** Characteristics of the study sample by trajectories of social communication deficits

Variable	Total sample (*n* = 804)	Social communication deficits trajectories		
		High (*n* = 80)	Low (*n* = 724)	*p* value for difference
Sex (*n* = 804): % boys	48.9	58.8	47.7	.059
Risk exposures				
Cumulative risk (*n* = 804): score	−0.31 (0.72)	−0.30 (0.91)	−0.31 (0.72)	.847
Maternal smoking (*n* = 798): % yes	12.2	13.9	11.8	.586
Child characteristics				
Conduct problems (*n* = 793): score	−0.30 (1.26)	1.34 (1.64)	−0.43 (1.04)	<.001
Hyperactivity/inattention (*n* = 792): score	−0.48 (2.53)	1.80 (3.11)	−0.56 (2.32)	<.001
Emotional difficulties (*n* = 792): score	−0.44 (1.26)	0.06 (2.05)	−0.47 (1.20)	.001
Peer problems (*n* = 793): score	−0.38 (1.02)	0.13 (2.02)	−0.45 (0.94)	<.001
IQ (*n* = 768)	107.32 ± 15.61	103.95 ± 19.68	107.70 ± 15.04	.215

*Note:* Values represent median (interquartile range) or mean ± SD unless otherwise specified; *p* values are derived from chi-square tests (categorical variables) or Mann–Whitney–Wilcoxon tests (continuous variables).

**Table 2.** DNA methylation loci at birth that prospectively associate with social communication deficits (SCD) trajectories

Probe	Gene	Chr	Genomic location	Position	*F*	*p*	FDR (*q* value)	Mean ± SD		% Difference	mQTL
								High SCD trajectory (*n* = 80)	Low SCD trajectory (*n* = 724)		
cg23234820	*A4GALT*	22	Body	43089041	27.11	2.54E^−07^	0.048	0.79 ± 0.06	0.82 ± 0.05	3	–
cg06825512	*APCDD1*	18	TSS1500	10453969	26.64	3.21E^−07^	0.048	0.51 ± 0.08	0.47 ± 0.07	4	–
cg04112471	*SPSB4*	3	5’UTR	140783430	26.42	3.58E^−07^	0.048	0.76 ± 0.07	0.80 ± 0.05	4	–

*Note:* Genomic location refers to the gene body, transcription start sites (TSS1500), and untranslated regions (5′UTR). Chr = chromosome; *F* = F-statistic for ANOVA; *p* = uncorrected *p* value; *q* = FDR-corrected value; mQTL = methylation quantitative trait loci; FDR = false discovery rate.

**Table 3.** Associations of differentially methylated probes with risk exposures and child characteristics

	Differentially methylated probes					
	cg23234820		cg06825512		cg04112471	
	β	*p* value	β	*p* value	β	*p* value
Risk exposures						
Cumulative risk: score	−0.04	.2977	0.06	.0962	−0.06	.0777
Maternal smoking: yes vs. no	−0.01	.7693	0.02	.5632	−0.05	.1461
Child characteristics						
IQ	0.00	.9775	0.00	.9943	0.03	.4446
Mental health symptoms: scores						
Conduct problems	−0.09	.0127	0.12	.0004	−0.09	.0063
Hyperactivity/inattention	−0.07	.0675	0.00	.3369	−0.12	.0006
Emotional difficulties	−0.04	.2566	0.02	.6021	0.00	.8929
Peer problems	−0.10	.0053	0.01	.8632	−0.03	.3574
SCD trajectories: low vs. high	−0.18	2.54E^−07^	0.17	3.21E^−07^	−0.17	3.58E^−07^

*Note:* Standardized regression coefficients and *p* values are presented. SCD = social communication deficits.

**Table 4.** Replication in the Generation R Study of the differentially methylated probes identified in ALSPAC

Probe	Social communication deficits					
	ALSPAC (age 8–17 years)		Generation R Study (age 6 years)			
	Trajectories		Continuous		Categorical	
	β	*p* value	β	*p* value	β	*p* value
cg23234820	−0.18	2.54E-07	0.001	.9821	0.004	.8655
cg06825512	0.17	3.21E-07	0.017	.4750	0.014	.5589
cg04112471	−0.17	3.58E-07	−0.030	.3026	0.001	.9766

*Note:* Standardized regression coefficients and *p* values are presented.
