# Supplementary material for: Epigenetic profiling of social communication trajectories and co-occurring mental health problems: a prospective, methylome-wide association study
Source: Dev Psychopathol. Author manuscript; Available in PMC 2023 Feb 1. (PMC8622455; doi:10.1017/S0954579420001662)

Supplementary materials

Supplementary Information 1 : DNA methylation preprocessing

Table S1. Mental health symptom scores

Table S2. Trajectories of social communication deficits in the total ARIES sample (N = 947)

Supplementary Information 1

DNA methylation preprocessing

The protocol followed manufacturer instructions using the recommended alternative incubation conditions for use with Illumina Infinium arrays. Illumina HumanMethylation450 BeadChips (Illumina, San Diego, USA) were run following the manufacturer's protocol with no modifications and arrays were scanned using an Illumina iScan (software version 3.3.28). Initial quality control of data generated was conducted using GenomeStudio (version 2011.1) to determine the status of staining, extension, hybridization, target removal, bisulfite conversion, specificity, non-polymorphic and negative controls.

Samples were distributed across slides in a semi-random approach to minimise the potential relationship between batch effects and other variables. During the data generation process a wide range of batch variables were recorded in a purpose-built laboratory information management system (LIMS). The LIMS also reported QC metrics from the standard control probes on the 450k BeadChip for each sample back to the laboratory.

Table S1. Mental health symptom scores: descriptive statistics in the study sample (N=804) and factor loadings

Conduct problems

	N	Minimum	Maximum	Mean	SD	Factor loading
8 yrs	734	0	9	1,42	1,441	0.77
10 yrs	757	0	9	1,15	1,331	0.84
13 yrs	719	0	9	1,13	1,332	0.70

Hyperactivity/inattention

	N	Minimum	Maximum	Mean	SD	Factor loading
8 yrs	734	0	10	3,27	2,391	0.83
10 yrs	755	0	10	2,78	2,155	0.88
13 yrs	719	0	10	2,75	2,088	0.77

Emotional difficulties

	N	Minimum	Maximum	Mean	SD	Factor loading
8 yr	734	0	9	1,54	1,712	0.72
10 yr	756	0	10	1,32	1,588	0.80
13 yr	720	0	8	1,16	1,496	0.65

Peer problems

	N	Minimum	Maximum	Mean	SD	Factor loading
8 yr	734	0	9	1,23	1,502	0.74
10 yr	755	0	8	1,00	1,376	0.82
13 yr	719	0	10	1,04	1,488	0.67

Table S2. Trajectories of social communication deficits in the total ARIES sample (N = 947)

Descriptive statistics

Social communication deficits, scores

	N	*M*	*SD*
Age 8	872	2.65	3.37
Age 11	845	2.11	3.25
Age 14	830	2.43	3.44
Age 17	781	2.88	3.88

Figure 1: Latent profile model with 2 classes

Entropy = 0.955

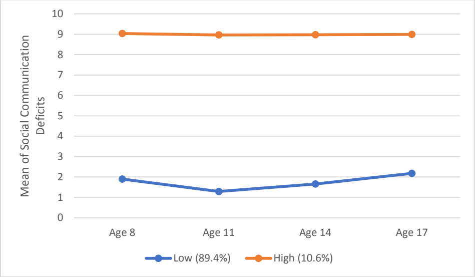

Figure 2: Latent profile model with 3 classes

Entropy = 0.916

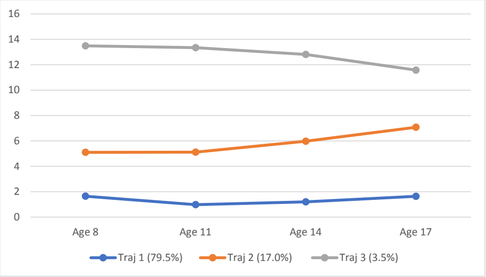

Figure 3: Latent profile model with 4 classes

Entropy = 0.904

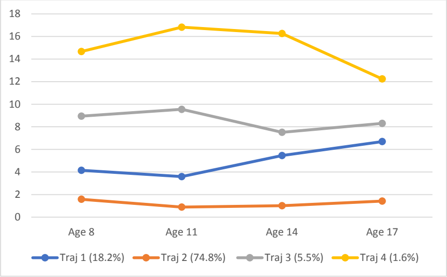

Supplement: 1 [file NIHMS1628665-supplement-1.pdf]
